# A Critical Analysis of Atoh7 (Math5) mRNA Splicing in the Developing Mouse Retina

**DOI:** 10.1371/journal.pone.0012315

**Published:** 2010-08-24

**Authors:** Lev Prasov, Nadean L. Brown, Tom Glaser

**Affiliations:** 1 Departments of Human Genetics and Internal Medicine, University of Michigan, Ann Arbor, Michigan, United States of America; 2 Division of Developmental Biology, Department of Pediatrics and Ophthalmology, Cincinnati Children's Research Foundation, University of Cincinnati School of Medicine, Cincinnati, Ohio, United States of America; Stanford University, United States of America

## Abstract

The *Math5* (*Atoh7*) gene is transiently expressed during retinogenesis by progenitors exiting mitosis, and is essential for ganglion cell (RGC) development. *Math5* contains a single exon, and its 1.7 kb mRNA encodes a 149-aa polypeptide. Mouse *Math5* mutants have essentially no RGCs or optic nerves. Given the importance of this gene in retinal development, we thoroughly investigated the possibility of *Math5* mRNA splicing by Northern blot, 3′RACE, RNase protection assays, and RT-PCR, using RNAs extracted from embryonic eyes and adult cerebellum, or transcribed *in vitro* from cDNA clones. Because *Math5* mRNA contains an elevated G+C content, we used graded concentrations of betaine, an isostabilizing agent that disrupts secondary structure. Although ∼10% of cerebellar *Math5* RNAs are spliced, truncating the polypeptide, our results show few, if any, spliced *Math5* transcripts exist in the developing retina (<1%). Rare deleted cDNAs do arise via RT-mediated RNA template switching *in vitro*, and are selectively amplified during PCR. These data differ starkly from a recent study (Kanadia and Cepko 2010), which concluded that the vast majority of *Math5* and other bHLH transcripts are spliced to generate noncoding RNAs. Our findings clarify the architecture of the *Math5* gene and its mechanism of action. These results have implications for all members of the bHLH gene family, for any gene that is alternatively spliced, and for the interpretation of all RT-PCR experiments.

## Introduction

The vertebrate retina develops from a single multipotent progenitor population, which gives rise to seven major cell types – rod and cone photoreceptors; amacrine, bipolar and horizontal interneurons; Muller glia; and retinal ganglion cells (RGCs) [1], [2]. These diverse cell types emerge from the mitotic progenitor pool in rough sequential order, with overlapping birthdates [3], [4]. RGCs are the first-born retinal cell type in every vertebrate examined [5]. These cells transmit all visual information from the eye to the brain, via their axons, which comprise the optic nerves. The gene network regulating retinogenesis is an active area of investigation.

An important clue toward understanding the mechanism of vertebrate retinal fate specification was the discovery of Math5 (Atoh7), a proneural basic-loop-helix (bHLH) transcription factor that is evolutionarily related to *Drosophila* Atonal and mouse Math1 (Atoh1) [6], [7]. The mouse *Math5* gene is expressed transiently in retinal cells exiting mitosis, from E11.5 until P0, in a pattern that is correlated with the onset of neurogenesis, and it is necessary for RGC fate specification. *Math5* mutant mice lack RGCs and optic nerves [8], [9], and have secondary defects in retinal vascularization [10] and circadian photoentrainment [11]. In zebrafish, the homologous *lakritz* mutation also causes RGC agenesis [12], and in humans, the *ATOH7* gene may be associated with congenital optic nerve disease [13]. Although the exact mechanism of *Math5* action remains unknown, it is thought to confer an RGC competence state on early retinal precursors [14], [15]. A number of potential target genes are misregulated in *Math5* mutant retinas [16]. Apart from the retina, expression domains have been defined in the hindbrain cochlear nucleus and cerebellum [17].

During our initial characterization of *Math5* [7], we identified multiple independent retinal cDNA clones, which were colinear and coextensive with mouse genomic DNA. The internal sequence and termini of these clones were consistent with a single-exon transcription unit.

In a recent provocative study, Kanadia and Cepko [18] report that the vast majority of *Math5* transcripts in embryonic mouse retinas are spliced, with donor and acceptor sites located in the 5′ and 3′ UTRs, such that the coding sequences are excised. This conclusion, which plainly differs from our previous studies [7], [13], was based largely on the size and abundance of particular RT-PCR products. Similar observations were reported for *Ngn3* (neurogenin, *Neurog3*), a related bHLH factor. If correct, these findings raise important questions regarding the origin, extent and function of noncoding (nc) bHLH-gene RNAs, which may integrate into larger gene regulatory networks during neural development [19], and suggest that abortive splicing may be utilized as a novel post-transcriptional mechanism to regulate bHLH gene expression. Given the importance of *Math5* for retinogenesis, the central role of bHLH factors in neuronal fate specification [20], and the possibility that functional coding and noncoding RNAs may be generated in the same orientation by alternative splicing of a single transcription unit [21], we have systematically evaluated *Math5* mRNA splicing in the developing retina, using RNA hybridization and RT-PCR methods adapted for the extreme G+C content of the transcript.

Our data strongly suggest that the apparently frequent splicing of *Math5* retinal mRNA is a technical artifact, resulting from: (1) profound secondary structure in the mRNA, promoting template switching during reverse transcription *in vitro*, (2) selective amplification of deleted products lacking the internal GC-rich segment; and (3) the existence of very rare mis-spliced molecules, representing less than one percent of *Math5* transcripts. Our results refine the structure of the *Math5* transcription unit, explore the concept of an intronless gene, and provide a cautionary lesson for PCR-based studies of RNA processing.

## Results

### *Math5* transcription unit, defined by cDNA clones, Northern and 3′RACE analysis

**Figure 1:**
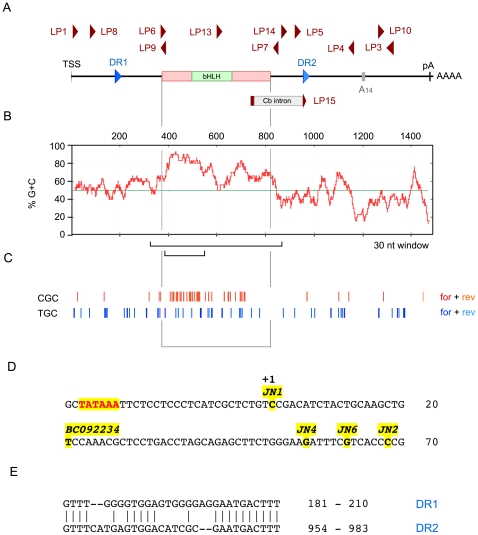
Anatomy of the *Math5* transcription unit. **A.** Gene map showing the major 1489 nt mRNA species; coding region (red box) and UTRs; direct repeats (DR); major polyA signal (pA) and internal A-rich segment (A_14_); cerebellar-specific intron (Cb); and PCR primers used in this study (dark red). LP15 spans the Cb intron junction. **B.** Plot showing elevated GC content (red) across the *Math5* coding region, compared to the average value (49.98%) for the mouse transcriptome (green) [83]. The 150 nt segment with >85% GC and the 536 nt fold encompassing the coding region are indicated (brackets). **C.** Concentration of polymerase-refractory YGC trinucleotides in the proximal coding region (both strands). **D.** Magnified view of the *Math5* promoter showing the TATAA box, transcription start site (TSS) and 5′ termini of cDNA clones [7], [13]. **E.** Sequence of UTR direct repeats.

**Figure 2:**
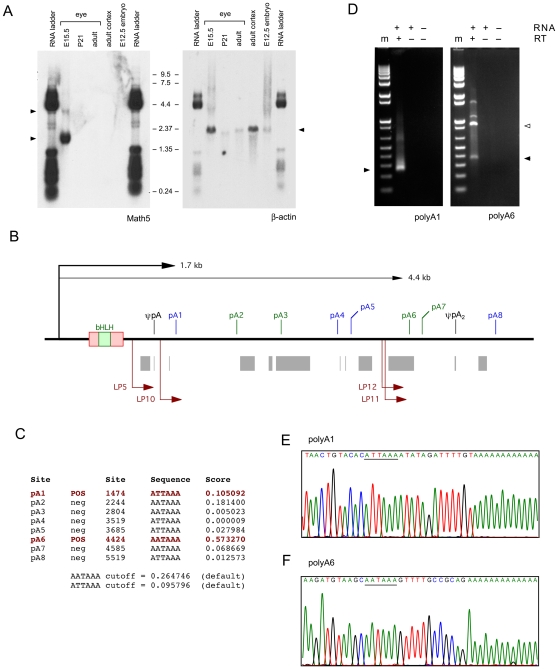
*Math5* messenger RNAs. **A.** Northern blot probed with 1.2 kb *Math5* (JN4C) and 1.1 kb β-actin cDNAs. Two *Math5* mRNAs are visible (left arrowheads), but no hybridizing RNA species is present in the 0.8–1.0 kb size range. The RNA size ladder cross-hybridized to vector DNA in the plasmid probes. **B.** Map of the 3′UTR and flanking genomic DNA (6 kb), showing eight potential polyA signals ATTAAA (blue) and AATAAA (green); the internal A_14_ priming site in the UTR (ψpA); interspersed repeats (gray); and the nested 3′RACE primers (dark red) for pA1 and pA6 sites, which have the most favorable sequence context. Clones JN2 and BC092234 terminate at pA1, whereas cDNAs JN1, JN4 and JN6 terminate at ψpA [7], [13]. ψpA_2_ marks an A-rich genomic site captured in the pA6 assay. **C.** polyADQ scores for all potential pA sites, calculated using human genome parameters [22]. Only pA1 and pA6 have scores above threshold. **D.** Embryonic eye RT-PCRs with 260 bp and 365 bp 3′RACE products (arrowheads) showing utilization of pA1 and pA6 sites. The 900 bp product was primed from ψpA_2_ (open arrowhead). m, marker (1 kb-plus ladder); RT, reverse transcriptase. **E.** Sequence of pA1 RACE products originating from the 1.7 kb *Math5* mRNA. **F.** Sequence of pA6 RACE products originating from the 4.4 kb *Math5* mRNA.

During our initial characterization of *Math5* [7], we identified four independent retinal cDNA clones, which were colinear with mouse genomic DNA (Genbank accession no. AF418923). The 5′ and 3′ termini, and internal sequences were consistent with RNA hybridization data suggesting a single-exon transcription unit, with an initiation site 23 bp downstream from a TATAAA box and a polyadenylation (pA) site 669 bp downstream from the TAA stop codon, giving 1.7 kb as the predicted size for polyA+ *Math5* mRNA (Figure 1a,d). This major *Math5* transcript was detected by Northern blot analysis of E15.5 mRNA with an 1155 bp radiolabeled cDNA probe (JN4C) that includes 318 bp 5′UTR, 447 bp coding sequence (CDS) and 390 bp 3′ UTR (Figure 2a). A second, less abundant 4.4 kb transcript was also detected at this age, which is close to the peak time-point for *Math5* expression during embryogenesis [15]. Careful inspection of the autoradiogram, in relation to the RNA size markers, revealed no smaller *Math5* transcripts, particularly in the 0.8–1.0 kb size range expected for spliced isoforms lacking the coding region. This pattern resembles Northern data obtained by Kanadia and Cepko with UTR probes (*cf.* Figure 1f and 1f'), but appears inverted compared to the unsized blot hybridized with a CDS probe in their report (*cf.* Figure 1f). We cannot explain this discrepancy.

To confirm our identification of the major *Math5* polyadenylation site [7] and define the 3′ terminus of the longer, 4.4 kb transcript, we first surveyed the 3′ *Math5* genomic region for favorable pA signals using the polyADQ weighted statistical algorithm [22]. Among eight potential pA sites downstream from the transcription start site (TSS), two had significant polyADQ scores (nos.1 and 6, Figure 2b,c), and these were consistent with the observed transcript sizes. We then looked for mRNAs terminating at pA1 and pA6 in parallel 3′RACE experiments [23], using E14.5 total eye RNA and nested primers positioned upstream of each site (Figure 2b,d). From the size and sequence of the products (Figure 2d–f), and our Northern data, we conclude that there are two principal *Math5* transcripts in the retina, 1.7 kb and 4.4 kb in length, and that both of these transcripts are *unspliced*. This interpretation is further supported by the curation of additional mouse cDNAs, represented as 56 expressed sequenced tags (ESTs) and two Genbank cDNAs in the NCBI database (Figure S1). Only two ESTs and one cDNA, originating from the adult cerebellum, appear to be authentic splice products (see below), and these do not correspond to the retinal isoforms reported by Kanadia and Cepko [18].

In addition to the coding region, *Math5* mRNA has three notable features relevant to this study (Figure 1a). First, the 5′ half is highly enriched in G+C nucleotides (Figure 1b), with >85% G+C content in the 150 nt segment spanning codons 7 to 57. *Math5* mRNA thus has the potential to form compact, thermodynamically stable secondary structures, owing to the third hydrogen bond in G–C pairs compared to A–U pairs, and the ability of guanine residues to interact with uracil in folded RNA [24]. The elevated G+C content is also predicted to affect folding of the (+) and (−) strand cDNA templates, compromising DNA polymerase processivity. Second, the 5′ segment of the gene is enriched for specific trinucleotide elements (Py-G-C) that are known to cause DNA polymerase pausing [25] (Figure 1c). These account for 15.7% of the trinucleotides in this segment (47 of 300, for both DNA strands), which is 1.73 fold higher than expected from mononucleotide frequencies. Third, mouse *Math5* mRNA contains 30-nucleotide imperfect direct repeats (DRs), located in the 5′ and 3′ UTRs (Figure 1a,e). These UTR repeats are not conserved among mammalian *ATOH7* mRNAs.

### Sensitivity of *Math5* PCR to template folding *in vitro*

**Figure 3:**
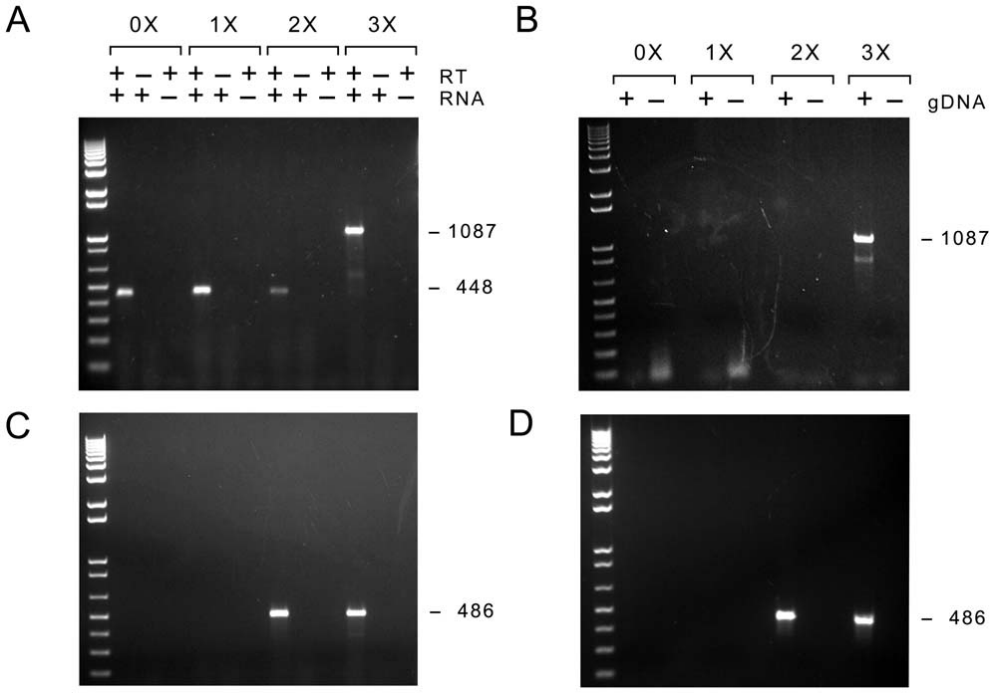
*Math5* embryonic eye RT-PCRs with increasing amounts of betaine. **A.** Agarose gel showing cDNA products amplified from DNase-treated E14.5 eye RNA with UTR primers LP8 and LP4 in the presence of 0X, 1X, 2X and 3X Masteramp™. When the betaine concentration was increased, only the full-length 1087 bp *Math5* cDNA product was visible; the 448 bp ECO product was absent. No amplimers were observed in the absence (−) of RNA template or RT enzyme. The identity of all PCR products was verified by sequencing. **B.** Similar PCR with a mouse genomic DNA template, showing amplification of the identical full-length 1087 bp product. **C,D.** Parallel PCRs were performed using internal primers LP6 and LP7. A single 486 bp *Math5* product was amplified from cDNA or gDNA in 2–3X Masteramp™.

Our Northern analysis, screening of cDNA libraries, and analysis of ESTs contrasts starkly with the abundant, heterogeneous splicing recently reported for the *Math5* gene [18]. As a first step to resolve this difference, we performed a series of RT-PCR experiments using the same primers (LP8 and LP4, Figure 1a and Table S1) and similar conditions (Table S2) as these authors. Using a thermostable reverse transcriptase (RT) formulation (Transcriptor™, Roche), E14.5 total mouse retinal RNA as template, and primers located in the 5′ and 3′ UTRs, we amplified a single 448 bp product (Figure 3a) with the same sequence as the ECO cDNA reported by Kanadia and Cepko [18] (Figure 3c), thus technically reproducing their primary observation. In this cDNA, a 639 bp segment encompassing the entire *Math5* coding region has been deleted. The 3′ breakpoint abuts the 3′UTR direct repeat.

The extremely high G+C content of the 5′ half of the deleted segment (Figure 1b) creates the potential for the RNA to form stable secondary structures, which could impede the procession of reverse transcriptase (RT) and DNA polymerases. Given our previous experience working with *Math5*, we repeated this PCR, replacing the water in the reaction mixture with 0 to 3X Masteramp™ (Epicentre). This is functionally equivalent to 0 to 1.0 M betaine (N,N,N-trimethyl glycine) [not shown], which is the principal ingredient in this additive [25], [26], [27]. In these reactions, betaine interacts with DNA as an isostabilizing agent, equalizing the free energies of A–T and G–C pairs by increasing hydration of the minor groove and flexibility of the double helix [28], [29]. It thus melts secondary structures, allowing DNA polymerases to extend through GC-rich segments [25], [26], [27]. In our experience, ≥1 M betaine is required to reliably amplify across the 5′ coding sequences of mouse or human *ATOH7*, even when cloned cDNA is used as a template; and relatively high concentrations (∼2 M) are tolerated in the PCR. Moreover, in the absence of betaine, we have observed numerous PCR-generated deletions of *Math5* sequences during molecular cloning projects over several years (not shown).

As the concentration of betaine in the PCR was increased, the apparently spliced 448 bp ECO product vanished, and a strong 1087 bp product appeared, corresponding to full-length, unspliced *Math5* cDNA (Figure 3a). The identity of these molecules was verified by sequencing gel-purified PCR products and multiple pCR4-TOPO plasmid clones derived from the PCR products. The effect of betaine on the generation of the 448 bp product suggests that *Math5* splicing either does not occur in nature, within the developing retina, or is an extremely rare event. Indeed, under normal circumstances, the smaller product should have been significantly favored during the amplification steps, with or without betaine. However, since the ECO product cannot be generated by PCR from mouse genomic DNA (Figure 3b) [28], [29] and depends on RT, it must be represented in the initial first-strand cDNA pool, albeit at an extremely low level (see below). These molecules could have been generated from rogue, aberrantly spliced mRNAs or by RNA template-switching during the reverse transcription step. Regardless of their origin, these rare cDNA amplicons (448 bp, 52.7% GC) should have a large selective advantage over the full-length co-terminal cDNA (1087 bp, 60.1% GC) during subsequent cycles of PCR.

**Figure 4:**
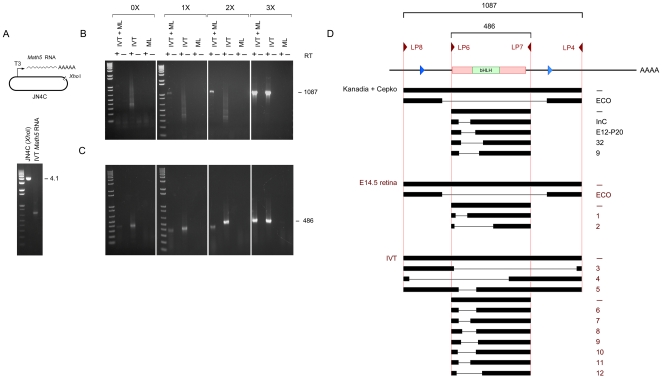
RT-PCRs of *Math5* RNA transcribed *in vitro*. **A.** Diagram and agarose gel showing linearized pJN4C and *Math5* sense RNA generated by T3 polymerase and treated with DNaseI. **B.** cDNA products amplified by RT-PCR from IVT-derived RNA with UTR primers LP8 and LP4. Only the full-length 1087 bp *Math5* cDNA product was amplified in the presence of 3X Masteramp (MA, indicated above brackets). In the absence of betaine, a variety of weak products were observed, with a heterogeneous deletion profile, reflecting a low level of RT template-switching. This background could be increased by using suboptimal PCR conditions or omitting the mouse liver RNA carrier. IVT, *in vitro* transcribed *Math5* RNA (10 ng); ML, mouse liver RNA (3 µg). **C.** Similar RT-PCRs performed using internal primers LP6 and LP7. Only the expected 486 bp cDNA was amplified in 3X MA, while spurious products were amplified at lower MA concentrations. The right three panels in B and C represent adjacent lanes in the same gels, displayed separately for clarity. **D.** Alignment of lacunar cDNAs generated from IVT or E14.5 eye RNA templates. The deletion profile is comparable to the distribution reported by Kanadia and Cepko [18] (*cf.* Table S1 and Figure 1), using the same primer pairs with no precautions for GC secondary structure. The sequence of breakpoints is given in Table S3, with microhomology at the inferred sites of RT template-switching.

Similar experiments were performed with a second pair of primers (LP6 and LP7), which are separated by 486 bp in genomic DNA and flank the GC-rich segment (Figure 3c,d). In the absence of betaine, these primers did not amplify any product. However, when 2–3X Masteramp™ was included in the PCR, only the expected 486 bp amplimer was observed. When we extended the PCR beyond 35 cycles, preincubated the reaction at 25°C (“cold start”) or used crude *Taq* polymerase preparations in the absence of betaine, a heterogeneous group of deleted (lacunar) products was observed (not shown), with a size and sequence distribution (Figure 4d, Table S3) similar to that reported by Kanadia and Cepko.

### Deleted PCR products derived from RNAs transcribed *in vitro*

To determine the origin of the lacunar cDNAs, we performed parallel RT-PCR experiments on RNA templates derived by *in vitro* transcription (IVT). Full-length, sense *Math5* transcripts were synthesized *in vitro* using bacteriophage T3 RNA polymerase and a *Xho*I-cleaved pJN4C DNA template (Figure 4a). RT reactions were performed as before, with oligo dT-priming, and 0–20 ng of the *in vitro* RNA transcript as template, alone or diluted into 3 µg total mouse liver RNA. When the PCRs were performed in 3X Masteramp™, full-length 1087 bp and 486 bp products were amplified (Figure 4b,c), identical to those generated from E14.5 retinal RNA (Figure 3a,c). However, when the betaine was reduced or omitted, we observed a variety of smaller products, with a size distribution (Figure 4b–d) and sequence diversity (Table S3) similar to that reported by Kanadia and Cepko (2010, *cf.* Table S1), despite the absence of retinal RNA, spliceosomes or other eukaryotic cell components.

Because these products depend on reverse transcriptase, they must have arisen via RNA template-switching during the RT reaction [30], despite the use of a thermostable recombinant enzyme mixture with high fidelity, processivity and proofreading features [31], [32]. A similar origin seems likely for the majority of apparently spliced *Math5* cDNAs reported by Kanadia and Cepko (*cf.* Table S1). Indeed, most of the deleted products obtained here and in the previous paper (Table S3) contain 5–10 nt direct sequence homology at the junctions [33], and the majority of these do not conform to consensus splice sites. The only remaining explanation – that *Math5* encodes a nuclear self-splicing mRNA – lacks precedent [34]. A possible exception is the ECO cDNA product, which was amplified from embryonic retinal RNA *in less than 1M betaine* (Figure 3a) but not from IVT-derived material or genomic DNA.

### Critical evaluation of *Math5* splicing by competitive RT-PCR

**Figure 5:**
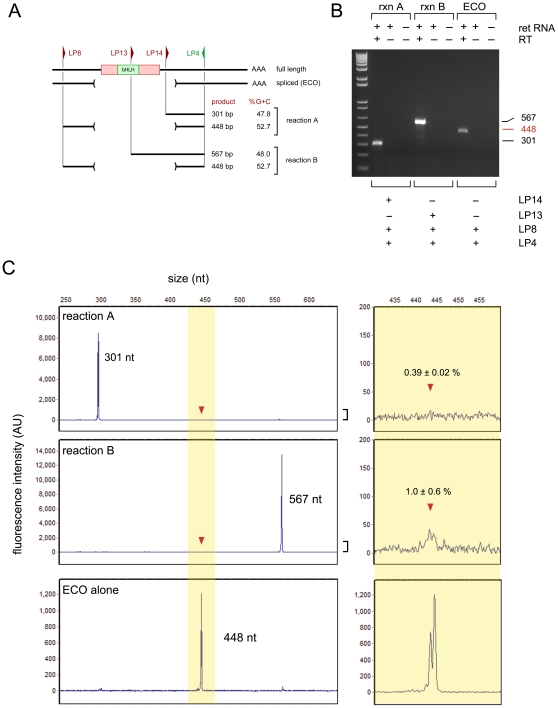
Triplex competitive RT-PCR assay to evaluate trace levels of *Math5* splicing in the embryonic retina. **A.** Diagram showing PCR strategy. The length and %G+C of competing amplicons are comparable. **B.** Agarose gel stained with ethidium bromide, showing only the unspliced *Math5* cDNA product in each assay. **C.** Capillary electrophoresis profiles showing triplex competitive RT-PCR products (**top panels**) and the ECO product amplified with duplex UTR primers in the presence of 1X MA (**bottom panel**). The common antisense primer (LP4) was end-labeled with 6-FAM. From the peak areas measured in replicate experiments and mixing controls, we estimate that the ECO product represents 0.4 to 1.0 percent of *Math5* mRNA in the embryonic retina, which is near the detection limit of this assay.

To further investigate *Math5* splicing *in vivo*, we directly compared the abundance of full*-*length and ECO (spliced) RNAs in competitive, triplex (3-primer) RT-PCR assays (Figure 5). Each reaction contained two alternative forward (sense strand) primers – one located in the 5′UTR and a second, internal primer in the 3′ coding region – plus a single reverse (antisense) primer located in the 3′UTR (Figure 5a). In this assay, the proportion of the two predicted products should reflect the relative abundance of the corresponding mRNAs in the E14.5 retina. The outer UTR primers and the resulting ECO product are identical to those reported by Kanadia and Cepko (see Table S1). For completeness, we performed two independent competitive RT-PCRs in parallel, with two different internal forward primers (LP13 and LP14), giving full-length products that were larger (567 bp) or smaller (301 bp) than the 448 bp ECO product, respectively. The spliced and unspliced products were also matched for G+C content (Figure 5a), so a direct comparison would be reliable. Moreover, these amplicons do not overlap the 5′ GC-rich segment of *Math5* that is refractory to RNA and DNA polymerase processivity. Only the full-length (unspliced) *Math5* products were detected in these experiments by ethidium bromide staining of agarose gels (Figure 5b). To consider this point more rigorously, and detect extremely rare spliced *Math5* mRNAs, we performed identical competitive RT-PCRs with a common 6-carboxyfluorescein (6-FAM) end-labeled reverse primer, and determined the molar ratio of spliced and unspliced products by fluorescence capillary electrophoresis (Figure 5c). We estimate that the ECO isoform represents less than 1.0% of *Math5* transcripts in the E14.5 eye. *Math5* splicing thus does not occur at significant levels in the developing mouse retina.

### Direct test of *Math5* splicing by nuclease protection (RPA)

To independently assess *Math5* mRNA splicing in the retina, we performed RNase protection assays [35]. The nuclease protection method was developed in the 1970s to demonstrate the existence of mRNA splicing [36], [37], [38]. Unlike PCR, nuclease protection assays do not depend on an exponential amplification process, which is highly sensitive to template secondary structure.

**Figure 6:**
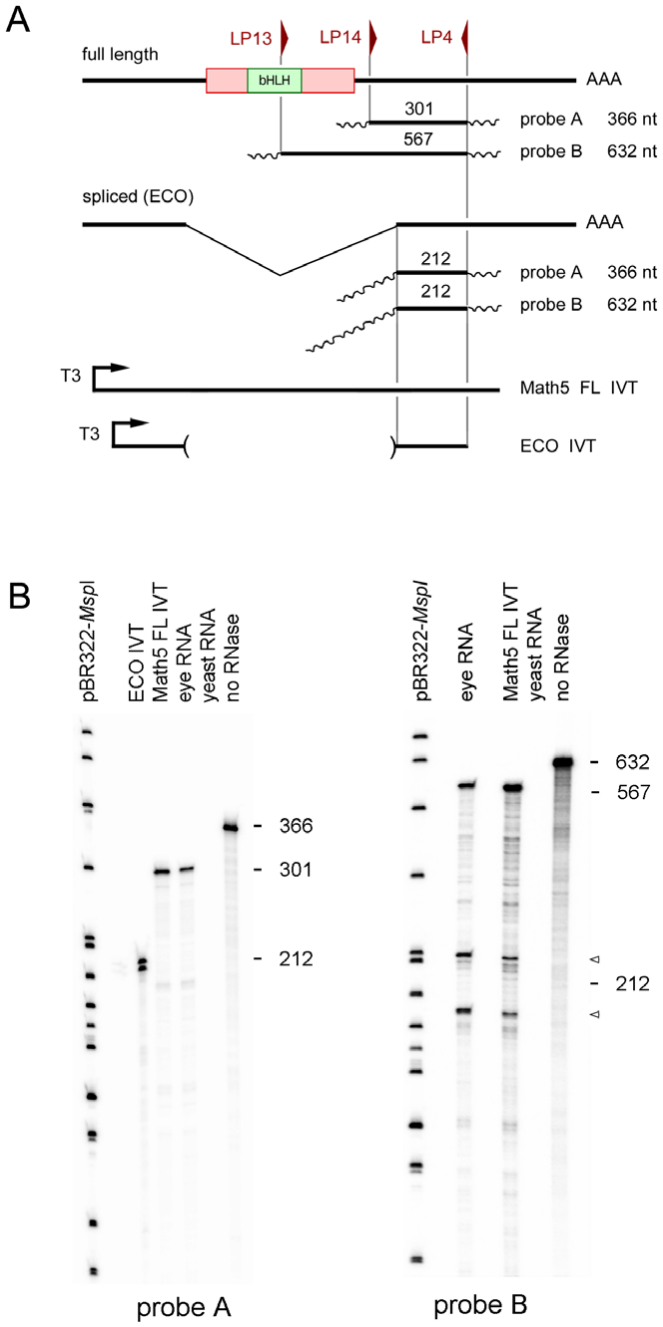
Ribonuclease protection assays. **A.** Diagram showing RPA strategy, with *Math5* cDNA, two different antisense cRNA probes, protected fragments expected for FL (full length, unspliced) and ECO (spliced) transcripts, and positive control RNAs generated by sense IVT reactions. **B.** Autoradiogram, showing undigested probes *A* and *B* (366 nt and 632 nt) and exclusively *unspliced* fragments protected by E14.5 eye RNA (567 nt and 301 nt). No fragment corresponding to the presumptive ECO transcript (212 nt) was protected by eye RNA using either cRNA probe, although a doublet of this size was protected by the ECO IVT positive control. Background fragments observed with probe *B* (arrowheads) are caused by intrinsic sensitivity of the cRNA-mRNA duplex to RNase cleavage at particular sites and were also present in the full length IVT positive control. The probe (no RNase) and IVT controls were diluted 20- and 10-fold respectively, compared to the E14.5 eye RNA hybridization lanes.

To evaluate the ratio of spliced and unspliced *Math5* transcripts, we hybridized total eye RNA from E14.5 embryos, in parallel, with a molar excess of two ^32^P-labeled antisense RNAs (Figure 6a). These cRNAs were prepared by *in vitro* transcription of two cDNA clones derived from unspliced 301 bp (A) and 567 bp (B) competitive RT-PCR products (Figure 5b). After hybridization and RNase digestion, surviving probe RNA molecules were resolved by polyacrylamide gel electrophoresis (Figure 6b). Probes A and B were protected by full-length *Math5* mRNA in the embryonic eye, giving 301 nt and 567 nt digestion products. No hybridizing fragments were detected at the size predicted for ECO mRNA (212 nt). The absence of smaller protected fragments in this sensitive assay further indicates that the *Math5* coding segment is not significantly spliced in the embryonic eye.

### Coding potential of lacunar *Math5* RNAs

In addition to the ECO product, which lacks the entire coding region, multiple mRNAs were proposed to originate from *Math5* primary transcripts via alternative splicing [18]. In some cases, these contain partial open reading frames and were predicted to encode shorter Math5 isoforms. To test this hypothesis, the authors used commercial Math5 peptide antisera to probe extracts from cells transfected with various splice products (*cf.* Figure 1h). We independently tested the reactivity of Math5 antibodies to mouse and human proteins expressed at high levels in transfected NIH3T3 cells, by Western blotting, and to retinal sections from wild-type and *Math5* mutant embryos (Figure S2), following standard precepts [39], [40]. We were unable to detect mouse Math5 polypeptide with any of these reagents, including the Abcam antisera (ab13536) used by Kanadia and Cepko [18].

### Spliced *Math5* transcripts in the cerebellum

During our previous characterization of *Math5*, we noted expression in the developing hindbrain and cerebellum, including a single hybridizing *Math5* mRNA detected by Northern analysis [17]. This 1.7 kb mRNA was consistent with the size of embryonic retinal transcripts (Figure 2a). However, three out of six *Math5* clones derived from adult mouse brain RNA in the NCBI database are apparently spliced, cerebellar (Cb) ESTs BY705389 and AV030226, and cDNA AK005214 (Figure S1a). These Cb isoforms are missing 199 nucleotides, and consequently are predicted to encode a truncated polypeptide in which the 22 terminal amino acids of Math5 (VDPEPYGQRLFGFQPEPFPMAS) are replaced by 2 residues (VS). Although the C-terminal amino acids are moderately conserved among amniotes (Figure S3c), the donor splice sites are not.

To evaluate *Math5* splicing in the cerebellum, we performed binary (2-primer) and competitive RT-PCR experiments with total adult cerebellar RNA as template (Figure S3). In the binary PCR, the primers flanked the putative 199 bp intron, giving 567 bp (unspliced) or 368 bp (spliced) products (Figure S3d). In the triplex PCR, the two forward primers were located inside the intron and spanning the exon junction (to amplify unspliced and spliced products respectively), and the common reverse primer was end-labeled (Figure S3e,f). The reactions involved the terminal portion of the *Math5* coding region and 3′UTR, and the products were similar in size (301 vs. 228 bp) and G+C content (47.8 vs. 52.2%). In contrast to the embryonic retina, we observed a moderate level of alternative mRNA splicing in the adult cerebellum, involving 11±2% of *Math5* transcripts. The major (1.7 kb) and minor (1.5 kb) cerebellar splice forms were not previously resolved in Northern blots [17], presumably because of the difference in abundance, and the effect of polyA tail heterogeneity (with an expected mean length of 250 adenosines, [41]. This shorter isoform was not detected in the embryonic retina (Figure S3d).

## Discussion

**Figure 7:**
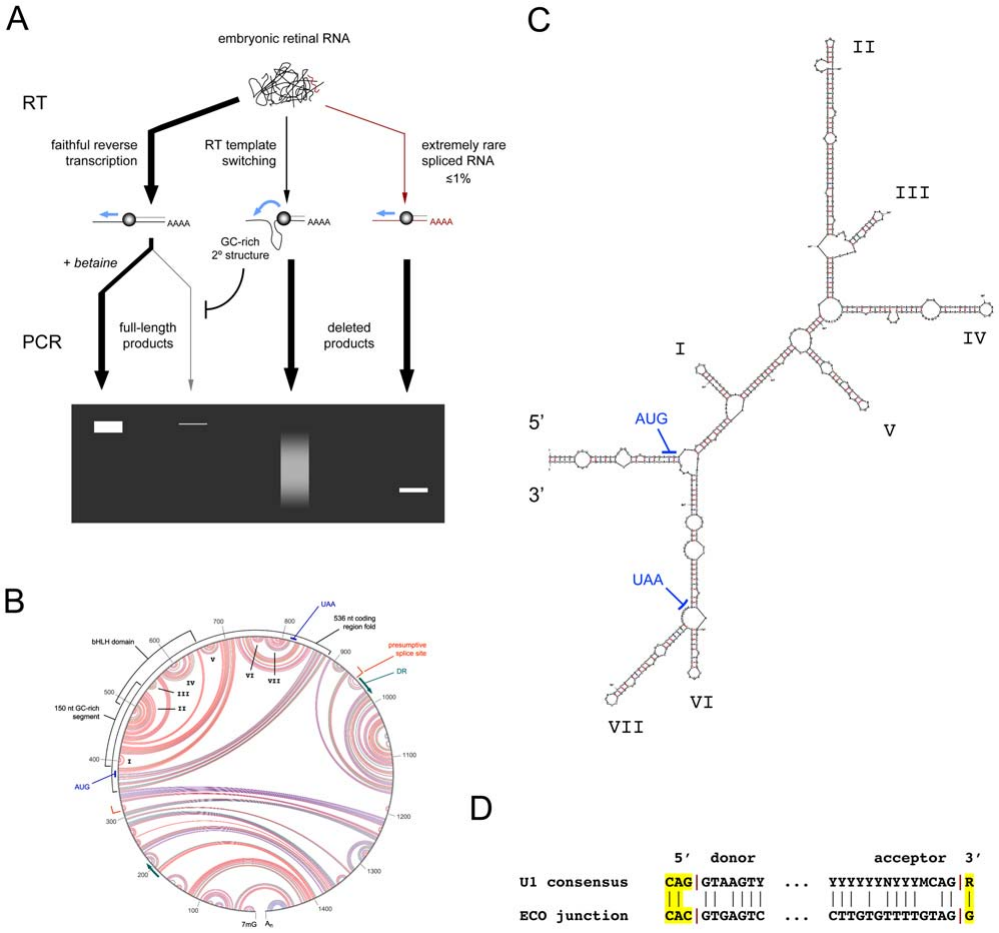
Model explaining the observed results. **A.** Diagram showing the likely origin of heterogeneous deleted *Math5* cDNAs, through combined effects of RT template switching, trace levels of aberrantly spliced ECO mRNA, and powerful PCR selection favoring deletion of GC-rich coding sequences. **B.** Secondary structure predicted for the major 1489 nt Math5 mRNA. This M-fold circle diagram, generated by free energy (ΔG) minimization, is magnified in Figure S5. Red, blue and green arc lines indicate G–C, A–U and A–G base pairs. The coding region, DRs and presumptive ECO splice sites are labeled. The 150 nt segment described in the text with >85% G+C, and the segment expanded in panel C are marked. **C.** Stem-loop diagram showing the 536 nt fold that encompasses the *Math5* CDS with lowest free energy (ΔG = −258 kcal/mol) and T_m_≥82°C. The major structural features in panels B and C are labeled alike. **D.** Junctional sequences for the ECO product with presumptive splice sites, compared to the U1 consensus.

We have critically defined the transcriptional anatomy of the *Math5* gene, and characterized alternatively spliced mRNAs. In contrast to the adult cerebellum, *Math5* mRNA is not significantly spliced in the developing retina. This conclusion is supported by six independent lines of evidence: (1) Northern analysis; (2) RT-PCR analysis of natural RNAs in the presence of graded betaine concentrations; (3) PCR of IVT-derived RNAs; (4) triplex competitive RT-PCR; (5) EST informatics; and (6) ribonuclease protection assays. Our findings differ sharply from the recent report of Kanadia and Cepko [18]. Three major factors contribute to the technical artifacts observed by these authors: (1) intense secondary structure in the >85% GC-rich segment of *Math5* RNA and cDNA, which blocks the progression of polymerase enzymes, creating a powerful negative selection; (2) RT template switching *in vitro*; and (3) the existence of a vanishingly small population of aberrantly spliced *Math5* mRNAs (Figure 7a). In view of these results, further investigation of *Ngn3* splicing may be warranted (Figure S4).

The GC-rich coding segment of *Math5* (Figure 1b) evidently forms a “Gordian knot” of secondary structure (Figure 7b,c), so dense that it favors the amplification of minor cDNA products, representing less than 1% of *Math5* molecules. G+C sequence bias is a well known problem in cDNA profiling studies [42], [43]. The folded hairpin structure of *Math5* mRNA is relaxed in the presence of betaine. *In vivo*, local melting is presumably catalyzed by DNA- and RNA-binding proteins, allowing *Math5* replication, transcription and translation. However, the tight RNA secondary structure may have consequences for Math5 protein expression. For example, translation may require specific mRNA unwinding activity, creating another potential mode of post-transcriptional regulation [44]. Indeed, mRNA hairpins are known to impede ribosome elongation [45] and G+C content is inversely correlated with translation efficiency [46]. If translation of the GC-rich *Math5* mRNA were hypersensitive to ribosome functional status, this may contribute to the disruption of RGC development in *Bst*/+ mice, which have a mutation in the *Rpl24* riboprotein gene and severe optic nerve hypoplasia [47].

On the basis of these results, we believe that the most likely explanation for the plethora of deleted *Math5* cDNAs (Figure 4) is RNA template-switching during the reverse transcriptase reaction, at points of sequence micro-homology (Figure 7a, Table S3) [33]. Indeed, RT polymerases are required to switch templates during normal retroviral replication, as part of the first and second transfer steps [48]. Aberrant switching *in vivo* can generate intramolecular deletions, and the frequency is positively correlated with the amount of RT pausing [49] and RNaseH activity [50]. In practice, template switching and related phenomena are well known hazards in PCR-based expression studies, and have been collectively termed “RT-facts” [30], [51], [52], [53], [54].

The process of eukaryotic splicing produces a variety of functional and nonproductive mRNAs during normal gene expression. While alternative splicing greatly extends the genetic repertoire [55], particularly in the nervous system [56], a significant fraction of Pol-II transcripts are mis-spliced, such that no protein or stable RNA species is synthesized, similar to the ECO isoform. Frequent errors include exon skipping, intron retention, and activation of cryptic splice sites. The resulting aberrant RNAs may outnumber correctly spliced mRNAs among initial spliceosomal products [57],[58]. For protein-coding genes with multiple exons, the majority of aberrant RNAs contain a premature truncation codon (PTC) and are degraded through the nonsense-mediated decay (NMD) pathway [59]. This is not generally possible for single-exon genes, which require distinct quality control mechanisms to eliminate defective mRNAs [60]. The intronless class represents 5–15% of mammalian genes [61], [62] and includes histones, GPCRs and many Zn finger, HMG, and bHLH domain transcription factors.

The process of splice site recognition is also far more complicated than the local pairing of 5′ and 3′ consensus sequences. It requires the *holo* definition of exon or intron elements in context, with integration of multiple splice enhancer and silencer effects [63], [64], [65], [66]. In this way, intronless genes may have selectively acquired sequence features that resist mRNA splicing [67], [68], [69]. Detailed sequence comparisons of intronless vs. intron-containing human genes have revealed differences in oligonucleotide frequencies and context-dependent codon biases [67]. The most striking characteristic of intronless genes in this analysis was the overrepresentation of GC-rich 4- to 6-mers, after correcting for base composition. The *Math5* cDNA matches this pattern extremely well (not shown), exhibiting sequence features that are characteristic of intronless genes. Moreover, the GGG triplet, which binds U1 snRNP as an intronic splice enhancer [70], [71], is depleted within the *Math5* coding region, despite the high G+C content. These global compositional features are not considered by the Spliceport algorithm that was used by Kanadia and Cepko to predict *Math5* splice sites. This web-based tool performs statistical analysis of *k*-mers in a 160 nt window surrounding putative donor and acceptor sites, based on human genome search data [72]. The analysis predicted the alternative Cb splice acceptor, which is utilized at low frequency in the adult cerebellum (FGA score  = 1.33); however, the Cb donor site was not identified and statistical support for donor sites in the *Math5* transcript was relatively low (max FGA score  = 0.26). Indeed, the mouse genome contains many more weak, potential splice sites than are actually utilized *in vivo*.

Among the numerous *Math5* species reported by Kanadia and Cepko, only one PCR product, termed ECO, is compatible with mRNA splicing. On the basis of our results, we believe this solitary cDNA is derived from an aberrantly spliced transcript, which has escaped normal quality control. First, the RNA encodes no protein and has no demonstrated function. In other contexts, long ncRNAs such as *Xist* and *Air*, have been shown to have regulatory roles [19], and a small number of bifunctional mRNAs have alternate coding and noncoding isoforms [21]. Second, the ECO isoform is very rare, representing less than 1% of *Math5* mRNA, and is thus unlikely to have a significant role in regulating *Math5* function or modulating retinal cell fate determination.

An intriguing result from our study is the discovery that 11% of mature *Math5* transcripts in the adult cerebellum are *bona fide* spliced mRNAs. These are predicted to encode a shorter Math5 protein, which lacks 20 amino acids from the C-terminus and may exhibit unique molecular properties (Figure S3). However, its function is not known, and *Math5* mutants have no overt cerebellar phenotype [17].

Despite the intriguing hypothesis advanced by Kanadia and Cepko, our results show splicing of *Math5* mRNA into noncoding isoforms does not occur in the developing retina at levels greater than 1% of transcripts. Further studies are needed to determine the exact mechanism of *Math5* action, how progenitors are transformed into neurons, and how noncoding RNAs, including microRNAs, may regulate *Math5* expression, RGC development, and the diversification of ganglion cell subtypes.

## Materials and Methods

### Plasmid clones and oligonucleotides

*Math5* clone pJN4C (accession nos. AF071223, AF418923) was derived from a neonatal C57BL/6 retinal cDNA library [7]. It contains 318 bp 5′UTR, 447 bp coding sequence (CDS) and 390 bp 3′UTR, and terminates at an A-rich stretch in the 3′UTR. Clones JN1 and JN2 extend 55 bp and 279 bp further in the 5′ and 3′ directions, respectively (Figure 1). Plasmid vector pCR4-TOPO (Invitrogen) was used for TA cloning of RT-PCR products, including the templates used for RPA probes. All custom PCR primers in this study are indicated in Figure 1a and listed in Table S1.

### Mice

The study protocol (09704) was approved by the University of Michigan Committee on the Use and Care of Animals (UCUCA). All mice were maintained in a specific-pathogen-free facility at the University of Michigan and experiments were performed in accordance with the provisions of the Animal Welfare Act, PHS Animal Welfare Policy, and NIH Guide for the Care and Use of Laboratory Animals.

### RNA

Total RNA was isolated from eyes or retinas dissected from CD-1 mouse embryos (ages E14.5 and E15.5) and adult tissues (eyes, cerebral cortex, cerebellum and liver) by the phenol-guanidinium-chloroform (Trizol) extraction method [73].

### Northern analysis

Ten µg total RNA from each tissue was resolved by formaldehyde-agarose gel electrophoresis and transferred to a 0.45 µm pore nitrocellulose membrane as described [74]. An RNA ladder in the 0.25–9.5 kb range (Gibco-BRL) was co-electrophoresed to accurately determine the size of hybridizing RNAs. After prehybridization, the membrane was probed successively with ^32^P-radiolabeled 1.2 kb *Math5* and 1.1 kb β-actin [75] mouse cDNAs, washed to 0.1X SSC 65°C stringency, and exposed to Kodak XAR film with an intensifying screen at −80°C for 16 hrs. The autoradiographic images were digitized using a flatbed scanner. The *Math5* probe was gel-purified from clone JN4C after digestion with *Xho*I and *Eco*RI, and was labeled to high specific activity with ^32^P-[α]-dCTP using the random hexamer (dN_6_) priming method [76].

### Reverse transcriptase (RT) and genomic PCRs

Total RNA from E14.5 or E15.5 mouse eyes (5 µg) or retinas (3 µg), adult cerebellum (5 µg), or adult liver (3 µg) was treated with 5 U DNaseI (Roche) for 15 min at 37°C in DNAse buffer (20 mM Tris-HCl, 2 mM MgCl_2_, 50 mM KCl). To stop the reaction, EDTA was added to 2 mM and the DNaseI was inactivated at 75°C for 10 min. RNAs were mixed with 500 ng oligo dT or 300 ng dN_6_ (Invitrogen) primer, denatured at 65°C for 10 min, and reverse-transcribed with 10 U Transcriptor™ High Fidelity RT (Roche) at 55°C for 1 hr. The 20 µL RT reactions contained 50 mM Tris-HCl pH 8.5, 30 mM KCl, 8 mM MgCl_2_, 5 mM DTT, 1 mM dNTPs, and 10 U RNase Inhibitor (Protector™, Roche). The RT was inactivated at 85°C for 5 min. The Transcriptor™ enzyme mixture has RNA-directed DNA polymerase, DNA-dependent DNA polymerase, helicase, RNaseH, and 3′→5′ exonuclease proofreading activities [31]. In the RT(−) controls, this enzyme mixture was replaced with nuclease-free water.

PCRs were performed using 1 µL of the cDNA reactions as template, in 1.5 mM MgCl_2_, 0.2 mM dNTPs, 20 mM Tris pH 8.4, 50 mM KCl, with 2 nM each primer and 2.5 U hot-start Platinum *Taq* polymerase or 0.5 U conventional *Taq* polymerase (Invitrogen). All PCRs were performed in 12-well strip tubes, in a 96-well MJ thermocycler with heated lid assembly, using specified primers and conditions (Table S1, S2). PCR products were separated by electrophoresis through 1.5% agarose gels, purified by membrane binding (Wizard SV, Promega) and sequenced or subcloned. Genomic PCRs were performed using 50 ng CD-1 mouse tail DNA.

To melt secondary structure, 10X Masteramp™ (Epicentre) was included in some PCRs, with a final fractional volume in the reaction mixture between 0.0 to 0.3 (v/v), designated 0X to 3X. Although the formulation of this additive is proprietary, equivalent results were obtained with 0.0 to 1.0 M betaine (Sigma B0300).

### *In vitro* transcription

Plasmid DNA (1 µg) from clones pJN4C [7] or pCR4-ECO was linearized with *Xho*I or *Not*I, respectively, and transcribed for 2 hr with 40 U bacteriophage T3 RNA polymerase (Roche), in a reaction containing 1 mM rNTPs, 40 mM Tris-HCl pH 8.0, 6 mM MgCl_2_, 10 mM DTT, 2 mM spermidine and 20 U RNase Inhibitor (Protector). The template was then digested with 20 U DNaseI for 1 hr at 37°C, and the resulting RNA was purified using Trizol (Invitrogen) and assessed by 1% agarose gel electrophoresis and UV absorbance (A_260_). The full length (FL) *Math5* IVT RNA product (10 ng) was mixed with DNaseI-treated mouse liver RNA (3 µg) or used directly (10–200 ng) for RT-PCRs.

### Triplex competitive RT-PCR assays

Retinal and cerebellar RT-PCRs [77] were performed in 1X MasterAmp™, with equal molar ratios of competing forward primers (1 nM) and a single fluorescent (6-FAM) reverse primer (LP4) as indicated (Table S2), which were matched for length and G+C content. Products were diluted to 1∶50 to 1∶200 in formamide and co-electrophoresed with GS-600 LIZ size marker in a 3730XL capillary DNA Analyzer (Applied Biosystems). The fluorescence intensity of each amplimer and the ratio of spliced to unspliced PCR products were calculated using GeneMarker (SoftGenetics), from the sum of major peaks in triplicate experiments.

### Rapid amplification of cDNA ends (3′ RACE)

First-strand cDNA synthesis was performed from retinal RNA as described above, using 10 pmol adapter primer (AP, Table S1). One µL of the cDNA reaction was then used to amplify 3′ terminal sequences using primers and conditions in Tables S1 and S2. To minimize spurious products from unrelated genes, a second round of PCR was performed using nested primers, following a conventional nested 3′RACE strategy [23].

### RNase protection assays (RPA)

RNase protection assays [35] were conducted using the RP-III kit (Ambion). Antisense cRNA probes were transcribed from PCR products A and B (Figure 5) cloned in pCR4-TOPO. One µg of each plasmid was digested with *Not*I and transcribed with T3 RNA polymerase as described above, except that 125 pmol (75 µCi) or 113 pmol (90 µCi) ^32^P-[α]-CTP was included in probe A and B reactions, respectively, with 200 pmol CTP and 10 nmol of ATP, GTP and TTP. This yielded 366 and 632 nt cRNA products with 301 nt (A) and 567 nt (B) direct sequence homology to *Math5*. Probes were purified by electrophoresis through denaturing 6% polyacrylamide gels and eluted for 3–4 hrs at 37°C. Ten µg of DNaseI-treated E14.5 eye RNA, yeast RNA (Ambion), or yeast RNA spiked with 10 ng *Math5* IVT product (ECO or FL) was precipitated in 2.5 M ammonium acetate 70% ethanol and resuspended in 8 µl hybridization buffer. The RNAs were hybridized with 2 µl probe A (8×10^4^ cpm) or probe B (1.2×10^5^ cpm) for 13 hr at 42°C, and digested with RNase A+T1 (1∶100) for 30 min at 37°C, and co-precipitated with glycogen and 5 µg yeast carrier RNA. Reactions were electrophoresed through 6% polyacrylamide denaturing gels (0.4 mm) in 6 M Urea and 0.5X TBE. dsDNA size markers were prepared by radiolabeling *Msp*I-digested pBR322 with ^32^P-[α]-dCTP and Klenow DNA polymerase. The dried gels were exposed to phosphor screens for 12–14 hrs and imaged using a Typhoon scanner (Molecular Dynamics) at 0.2 mm resolution. Yeast RNA controls were included ± RNase, to assess the probe integrity and the completeness of digestion.

### Informatics

Sequence alignments, G+C and antigenicity profiles, and PCR primer optimization were performed using MacVector (Accelrys) software and NCBI BLAST servers. *Math5* polyadenylation sites were predicted using the polyADQ [22] web server (rulai.cshl.org/tools/polyadq/). Scores were calculated for a 6.0 kb sequence extending from the transcription start site (Figure 1d), using default threshold values. RNA secondary structures were predicted by free-energy minimization [24], [78] using the M-fold web server (mfold.bioinfo.rpi.edu/). Expressed sequence tags (ESTs) for mouse and human bHLH cDNAs were accessed through the UCSC genome browser (genome.ucsc.edu/).

### Math5 antibodies

Commercial and custom antibodies to Math5 peptides and recombinant proteins are indicated in Figure S2 along with the immunogen, including the Abcam polyclonal reagent (ab13536) cited by Kanadia and Cepko [18]. Custom rabbit polyclonal sera were generated using internal (RCEQRGRDHP) or C-terminal (RLFGFQPEPFPMAS) Math5 peptide haptens coupled to KLH (keyhole limpet hemocyanin) via a cysteine thiol linkage (Research Genetics, Huntsville, AL), and were affinity purified.

### Cell transfection and Western analysis

NIH3T3 fibroblast cultures (ATCC, CRL-1658) were transfected with expression plasmid DNA (1 µg per 60 mm plate) for native or N-terminal 6xMyc-tagged versions of mouse or human ATOH7 proteins, or empty vector, using Fugene-6 reagent (Roche), with 0.1 µg pUS2-EGFP as an internal control. These plasmids were prepared by inserting ATOH7 coding regions from genomic phage, plasmid or BAC clones into pCS2 and pCS2MT vectors [79] and verifying the sequence. Mouse pCS2-Math5 and pCS2MT-Math5 plasmids were described previously [7]. After 48 hrs, cells were harvested in PBS with protease inhibitors (Complete™, Roche), lysed in RIPA buffer [80], sonicated, and centrifuged at 13,000×*g* for 15 min at 4°C. Soluble proteins were electrophoresed through NuPAGE Novex Bis-Tris 4–12% polyacrylamide gels (25 µg per lane), transferred to nitrocellulose membranes and stained with Ponceau S. Parallel Western blots were probed with rabbit polyclonal antisera to Math5 peptides (1∶200, Figure S2a), full-length human ATOH7 (D01P, 1∶500) or GFP (Abcam ab290, 1∶2500); or mouse anti-Myc monoclonal (9E10, Zymed, 1∶500); and the reactive proteins were visualized using HRP-conjugated anti-rabbit (NEN, 1∶5000) or mouse (GE, 1∶20,000) IgG secondary antibodies, enhanced chemiluminesence reagents (ECL-Plus, GE), and Kodak MS X-ray film.

### Immunostaining and RNA *in situ* hybridization

Mouse E15.5 embryo heads from wild-type and *Math5* knockout (*Atoh7* ^tm1Gla^) littermates [8] were fixed in 4% paraformaldehyde PBS for 1 hr at 4°C; processed through a 10–30% sucrose series in PBS; cryoembedded in OCT media (Tissue-Tek, Torrence, CA) and sectioned through the eyes at 5–10 µm. To thoroughly test antibody reactivity, we tried three different antigen unmasking protocols in parallel: 0.1 M Tris pH 9.5 at 95°C for 5 min; 0.05% trypsin at 37°C for 10 min; and 0.3% Triton X-100 0.1 M Tris pH 7.4 at 25°C for 10 min. Cryosections were then blocked and processed in TST milk as described [81]. Slides were incubated overnight at 25°C with a 1∶500 dilution of anti-Math5 peptide sera (Abcam no. ab13536, lot no. 610696), followed by a 1∶5000 dilution of Alexa594-conjugated goat anti-rabbit IgG secondary antibody (Molecular Probes). RNA *in situ* hybridization was performed on E15.5 embryonic retinas as described [82]. A digoxigenin-labeled antisense *Math5* cRNA probe spanning the 3′UTR and CDS was prepared from *Asc*I-digested plasmid pJN4C with T7 RNA polymerase, hybridized to retinal sections overnight, detected using an AP-conjugated sheep anti-DIG antibody (1∶2000, Roche), and visualized using NBT-BCIP histochemistry. Micrographs were imaged using a Zeiss Axioplan2 microscope, digital camera and Axiovision software.

## Supporting Information

Figure S1 *Math5* ESTs in the public domain. A. Diagram modified from the UCSC mouse genome browser (mm9 assembly, chr10:62,562,000–62,564,300) showing 56 *Math5* ESTs and 2 Genbank cDNAs (BC092234, AK005214), giving a total *n* = 58, with 52 derived from the embryonic retina. Forty-three of these retinal cDNAs cross the presumptive ECO junctions at the 5′ or 3′ side, and are thus informative for splicing (83%). Yet none originated from spliced mRNA. Of the remaining six, from adult brain RNA (red), two cerebellar ESTs and one cDNA were spliced at the Cb intron (yellow shading, see Figure S3). Nine 3′ ESTs out of 21 terminate at pA1; the remaining 12 were primed from ψpA. B. Comparable region of the human genome (hg19 assembly, chr10:69,992,300–69,990,000) showing one full-length Genbank cDNA and 7 unspliced ESTs.
(0.98 MB TIF)

Figure S2 Evaluation of Math5 antibodies. A. Diagram of the mouse Math5 protein, showing the antigenic index [84] and positions of immunogens used by various sources to prepare antibodies, as follows: *a,b* internal and C-terminal peptides (Glaser lab); *c*, ab13536 (Abcam); *d*, AB5694 (Chemicon); *e*, EB07972 (Everest); *f*, 1A5 (multiple vendors). The immunogens for D01P (Abnova) and MAb 1A5 were full-length or partial recombinant human proteins (gray); all others were based on the mouse polypeptide (blue). No immunogen was specified for ab78046 (Abcam). B. Immunoblots of NIH3T3 cells co-transfected in parallel with pUS2-EGFP and pCS2 expression plasmids for full-length mouse or human Math5 proteins ± six N-terminal Myc epitope tags, or empty pCS2 vector. Five identical blots were probed using antibodies with stated reactivity to mouse (ab13536, ab78046) or human (D01P) Math5; Myc or GFP. The predicted mass for native and 6xMyc mouse Math5 proteins is 16.9 and 27.0 kDa, respectively. Antibody D01P detected the human polypeptides, but not mouse. No other reagent tested was effective, including ab13536 (Abcam) [18], even when the Math5 proteins were massively overexpressed. C. Retinal sections from E15.5 embryos immunostained with ab13536 sera. The immunofluorescence pattern was identical between wild-type and *Math5* −/− eyes and is thus nonspecific [39], [40]. This pattern, which includes lens and RPE nuclei, does not fit the apical distribution of *Math5* mRNA in the neuroblastic retina. The *in situ* hybridization pattern of a *Math5* cRNA probe spanning the 3′UTR and CDS matches our previous reports [7], [85] and both panels provided by Kanadia and Cepko (*cf.* Figure 1j and 1j').
(1.96 MB TIF)

Figure S3 *Math5* splicing in the cerebellum. A. Diagram of alternative Cb intron, with PCR primers and products. B. Sequence of Cb splice junction, corresponding to nucleotides 3524 and 3724 in Genbank acc. AF418923. The acceptor site coincides with the ECO junction (Figure 7d). C. Spliced cerebellar mRNA encodes a truncated Math5 protein, with 20 fewer amino acids at the C-terminus. The deleted peptide has a similar sequence among amniotes, but the splice junction is not obviously conserved. D. Agarose gel showing spliced (368 bp) and unspliced (567 bp) RT-PCR products from the adult cerebellar RNA, but not from E14.5 retina. E. Triplex competitive RT-PCR showing spliced (228 bp) and unspliced (301 bp) products co-amplified from cerebellar cDNA (right lanes). In the duplex control with primers LP15 and LP4, only the Cb form (spliced) was amplified (left lanes). F. Capillary electrophoresis profiles showing the ratio of spliced (Cb) and unspliced (FL) transcripts in the triplex PCR (top), with Cb duplex product as a control (bottom). The common antisense primer LP4 was labeled with 6-FAM. Approximately 11±2 percent of *Math5* mRNAs are spliced at the Cb site in the adult cerebellum.
(2.89 MB TIF)

Figure S4 Splicing patterns in the mouse Atonal-related bHLH genes. A. Phylogram of mouse Ato proteins, based on maximum parsimony analysis of the bHLH domain across many taxa [13], [86]. B. Exon-intron organization of bHLH genes based on a survey of ESTs in the NCBI database [55], [87]. The eight mouse Ato homologs either have unitary exon structures, or a single intron located in the 5′ UTR. The Achaete-Scute homolog *Mash1* (*Ascl1*) has a single intron in the 3′ UTR. There is no obvious correlation between splicing patterns and locations in the mouse genome. MMU, mouse chromosome; ESTs, number of expressed sequence tags supporting the gene structure; *has minor alternative spliced product (Cb); **has overlapping intergenic and antisense RNAs. The intron of one spliced antisense EST (CF104925) for *Ngn3* (*Neurog3*) overlaps the 5′ UTR and coding sequence of the sense strand. This antisense RNA is predicted to co-amplify in the RT-PCR and may be mistaken for non-coding sense products.
(0.19 MB TIF)

Figure S5 Secondary structure for Math5 mRNA. This circle plot was generated by free energy minimization of the 1489 nt mRNA, and is enlarged from Figure 7. Red, blue and green arc lines indicate G–C, A–U and A–G base pairs. The coding region, DRs and presumptive ECO splice sites are labeled. The 150 nt segment with >85% G+C, and 536 nt segment spanning the CDS are marked. The CDS contains a high density of G–C base pairs (red arcs), which are deleted in rare, mis-spliced RNAs.
(1.33 MB TIF)

Table S1 Oligonucleotide primers in this study.
(0.03 MB PDF)

Table S2 PCR conditions in this study.
(0.03 MB PDF)

Table S3 DNA sequence flanking deletions in RT-PCR products.
(0.04 MB PDF)
