# Supplementary figures and images for: A Critical Analysis of Atoh7 (Math5) mRNA Splicing in the Developing Mouse Retina
Source: PLoS One. 2010 Aug 24;5(8):e12315. doi: 10.1371/journal.pone.0012315 (PMC2927423; doi:10.1371/journal.pone.0012315)

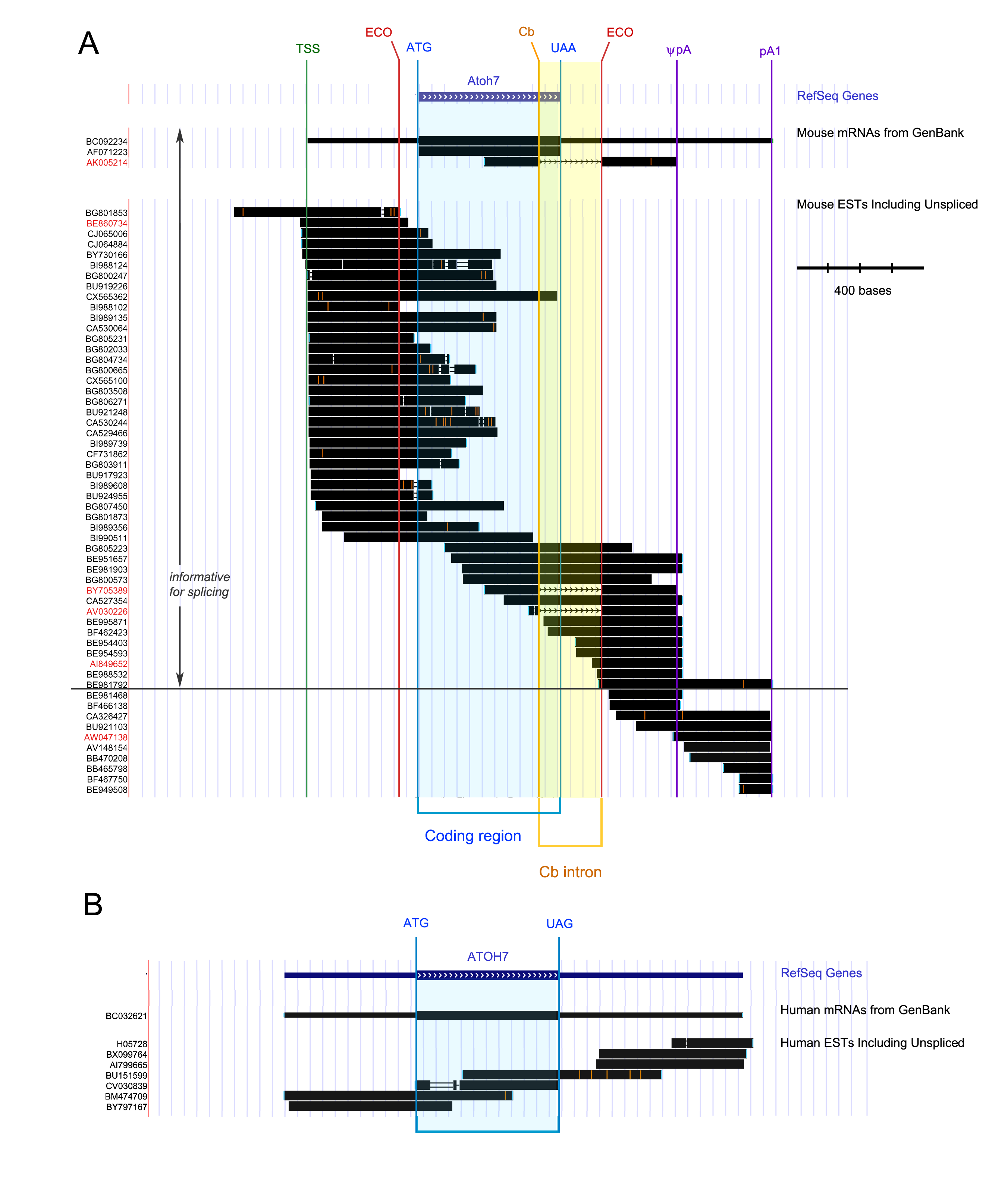

Supplement: Figure S1 — Math5 ESTs in the public domain. A. Diagram modified from the UCSC mouse genome browser (mm9 assembly, chr10:62,562,000–62,564,300) showing 56 Math5 ESTs and 2 Genbank cDNAs (BC092234, AK005214), giving a total n = 58, with 52 derived from the embryonic retina. Forty-three of these retinal cDNAs cross the presumptive ECO junctions at the 5′ or 3′ side, and are thus informative for splicing (83%). Yet none originated from spliced mRNA. Of the remaining six, from adult brain RNA (red), two cerebellar ESTs and one cDNA were spliced at the Cb intron (yellow shading, see Figure S3). Nine 3′ ESTs out of 21 terminate at pA1; the remaining 12 were primed from ψpA. B. Comparable region of the human genome (hg19 assembly, chr10:69,992,300–69,990,000) showing one full-length Genbank cDNA and 7 unspliced ESTs. (0.98 MB TIF) [file pone.0012315.s001.tif]

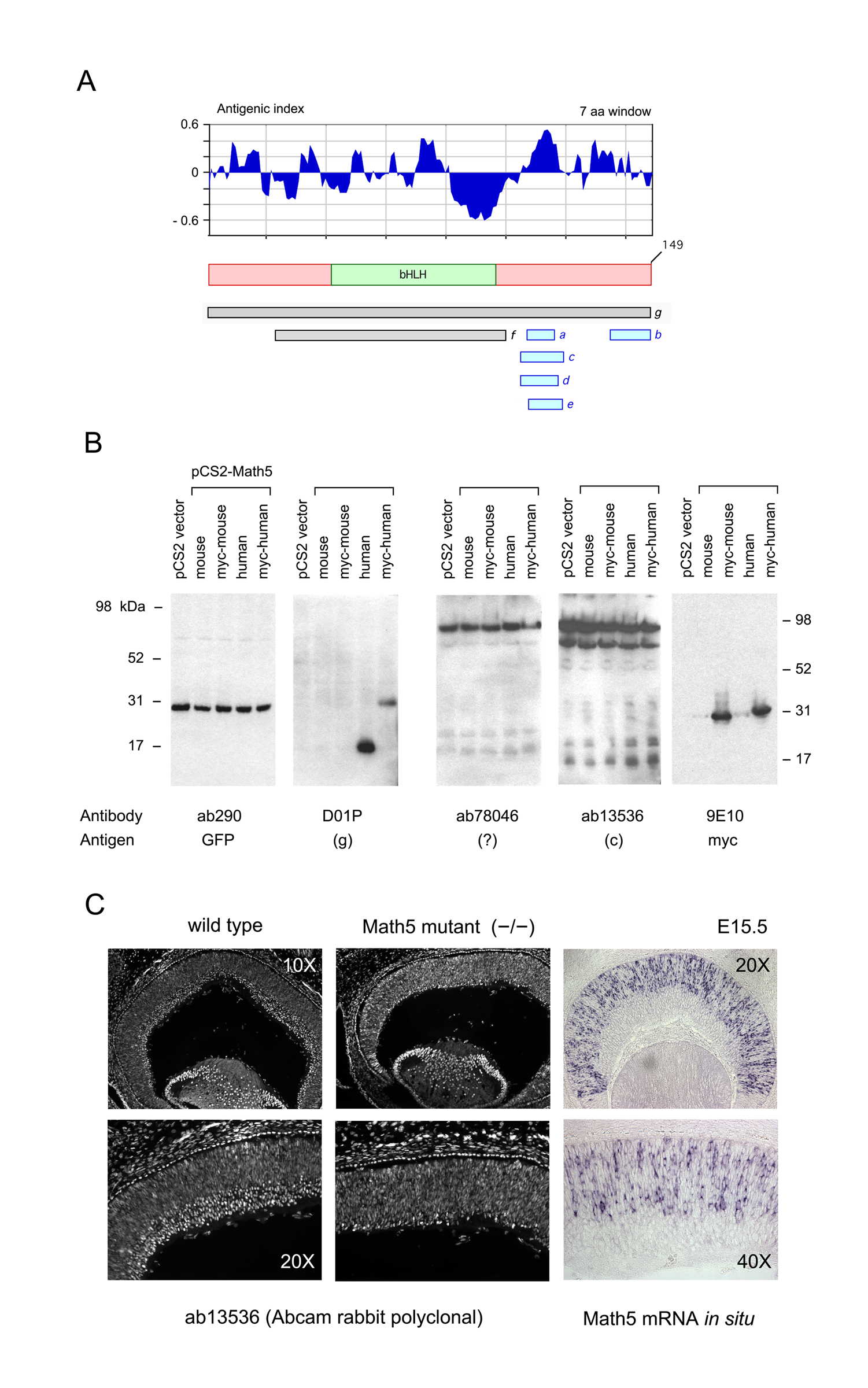

Supplement: Figure S2 — Evaluation of Math5 antibodies. A. Diagram of the mouse Math5 protein, showing the antigenic index [84] and positions of immunogens used by various sources to prepare antibodies, as follows: a,b internal and C-terminal peptides (Glaser lab); c, ab13536 (Abcam); d, AB5694 (Chemicon); e, EB07972 (Everest); f, 1A5 (multiple vendors). The immunogens for D01P (Abnova) and MAb 1A5 were full-length or partial recombinant human proteins (gray); all others were based on the mouse polypeptide (blue). No immunogen was specified for ab78046 (Abcam). B. Immunoblots of NIH3T3 cells co-transfected in parallel with pUS2-EGFP and pCS2 expression plasmids for full-length mouse or human Math5 proteins ± six N-terminal Myc epitope tags, or empty pCS2 vector. Five identical blots were probed using antibodies with stated reactivity to mouse (ab13536, ab78046) or human (D01P) Math5; Myc or GFP. The predicted mass for native and 6xMyc mouse Math5 proteins is 16.9 and 27.0 kDa, respectively. Antibody D01P detected the human polypeptides, but not mouse. No other reagent tested was effective, including ab13536 (Abcam) [18], even when the Math5 proteins were massively overexpressed. C. Retinal sections from E15.5 embryos immunostained with ab13536 sera. The immunofluorescence pattern was identical between wild-type and Math5 −/− eyes and is thus nonspecific [39], [40]. This pattern, which includes lens and RPE nuclei, does not fit the apical distribution of Math5 mRNA in the neuroblastic retina. The in situ hybridization pattern of a Math5 cRNA probe spanning the 3′UTR and CDS matches our previous reports [7], [85] and both panels provided by Kanadia and Cepko (cf. Figure 1j and 1j'). (1.96 MB TIF) [file pone.0012315.s002.tif]

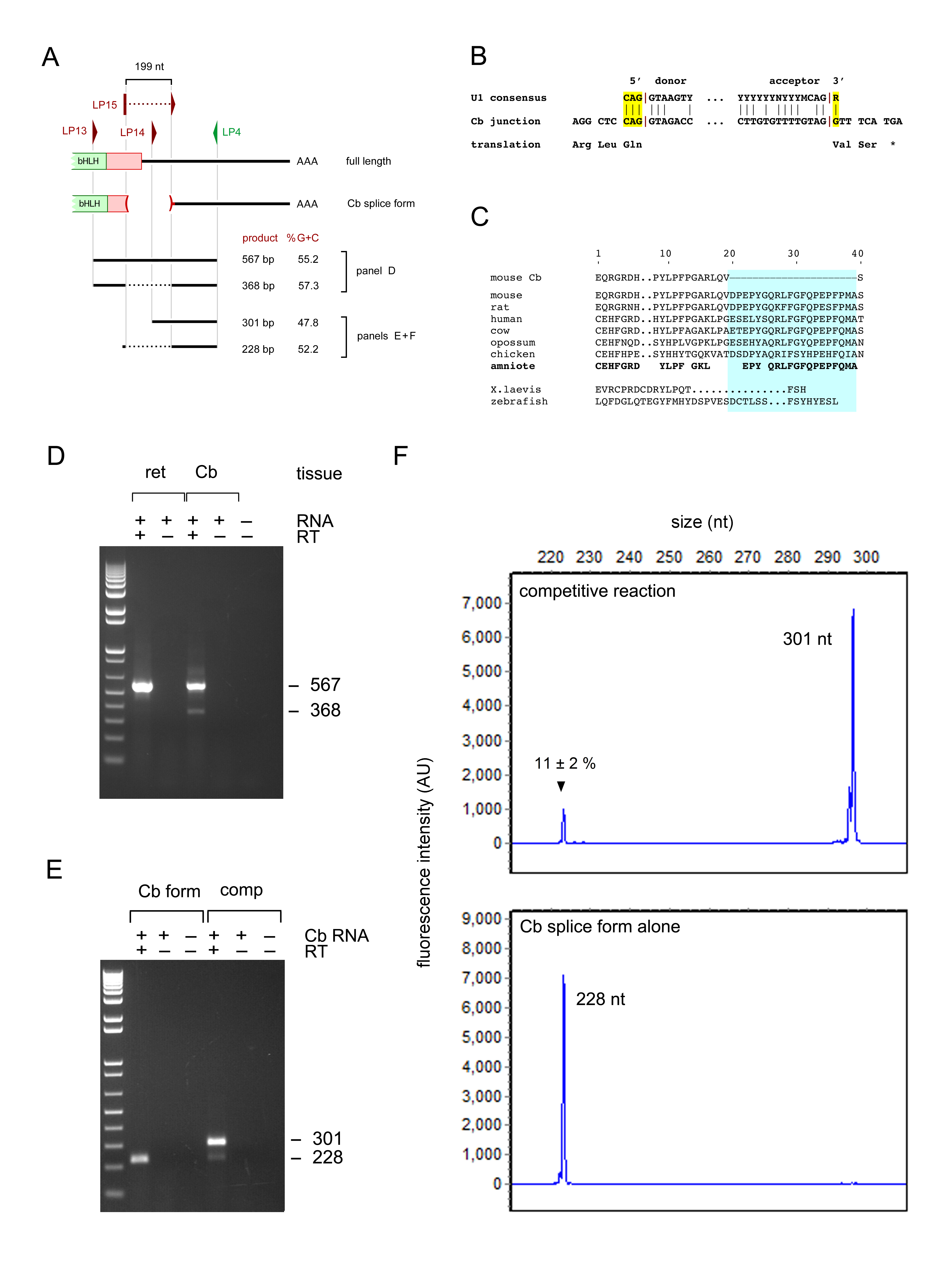

Supplement: Figure S3 — Math5 splicing in the cerebellum. A. Diagram of alternative Cb intron, with PCR primers and products. B. Sequence of Cb splice junction, corresponding to nucleotides 3524 and 3724 in Genbank acc. AF418923. The acceptor site coincides with the ECO junction (Figure 7d). C. Spliced cerebellar mRNA encodes a truncated Math5 protein, with 20 fewer amino acids at the C-terminus. The deleted peptide has a similar sequence among amniotes, but the splice junction is not obviously conserved. D. Agarose gel showing spliced (368 bp) and unspliced (567 bp) RT-PCR products from the adult cerebellar RNA, but not from E14.5 retina. E. Triplex competitive RT-PCR showing spliced (228 bp) and unspliced (301 bp) products co-amplified from cerebellar cDNA (right lanes). In the duplex control with primers LP15 and LP4, only the Cb form (spliced) was amplified (left lanes). F. Capillary electrophoresis profiles showing the ratio of spliced (Cb) and unspliced (FL) transcripts in the triplex PCR (top), with Cb duplex product as a control (bottom). The common antisense primer LP4 was labeled with 6-FAM. Approximately 11±2 percent of Math5 mRNAs are spliced at the Cb site in the adult cerebellum. (2.89 MB TIF) [file pone.0012315.s003.tif]

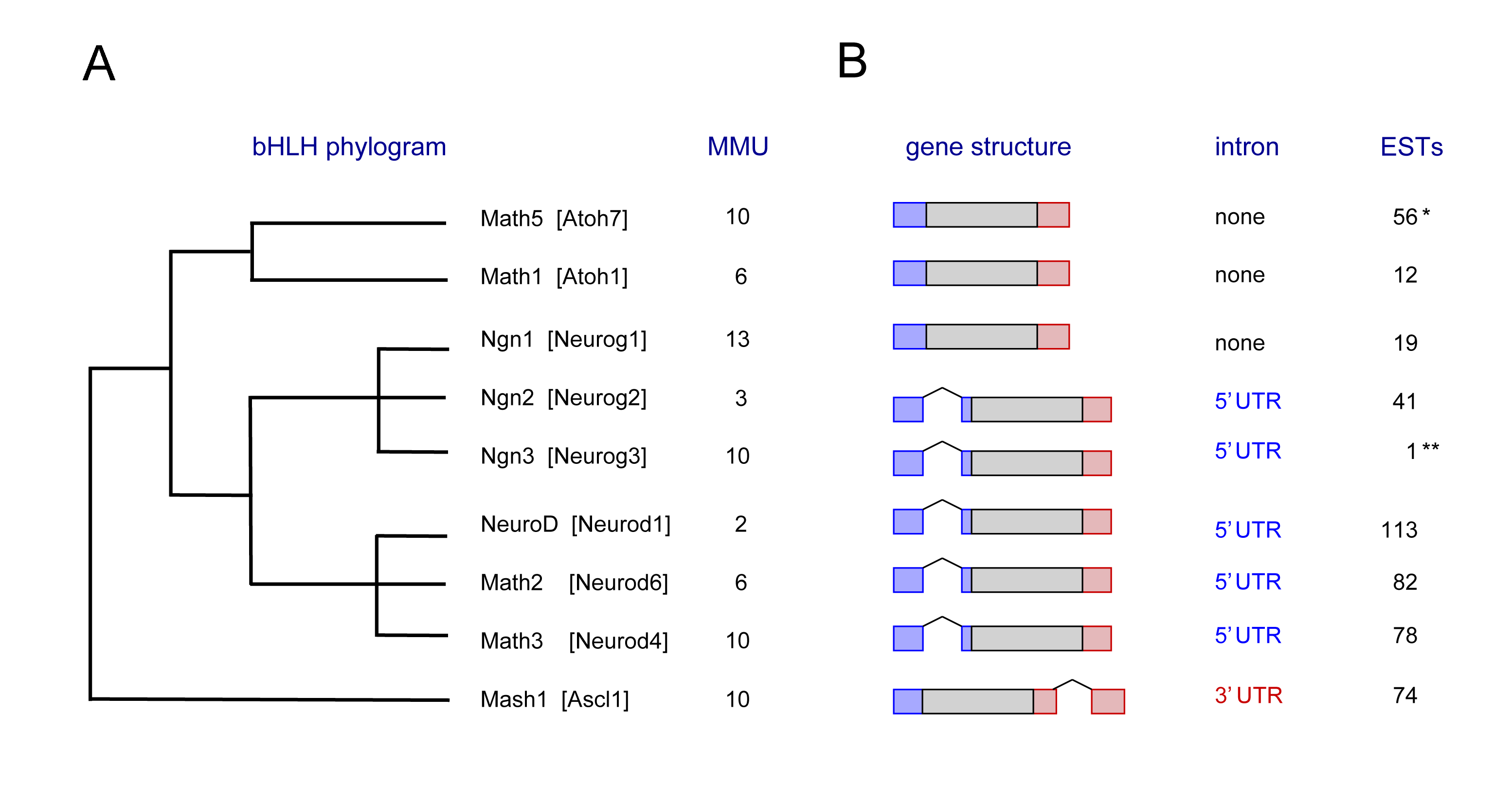

Supplement: Figure S4 — Splicing patterns in the mouse Atonal-related bHLH genes. A. Phylogram of mouse Ato proteins, based on maximum parsimony analysis of the bHLH domain across many taxa [13], [86]. B. Exon-intron organization of bHLH genes based on a survey of ESTs in the NCBI database [55], [87]. The eight mouse Ato homologs either have unitary exon structures, or a single intron located in the 5′ UTR. The Achaete-Scute homolog Mash1 (Ascl1) has a single intron in the 3′ UTR. There is no obvious correlation between splicing patterns and locations in the mouse genome. MMU, mouse chromosome; ESTs, number of expressed sequence tags supporting the gene structure; *has minor alternative spliced product (Cb); **has overlapping intergenic and antisense RNAs. The intron of one spliced antisense EST (CF104925) for Ngn3 (Neurog3) overlaps the 5′ UTR and coding sequence of the sense strand. This antisense RNA is predicted to co-amplify in the RT-PCR and may be mistaken for non-coding sense products. (0.19 MB TIF) [file pone.0012315.s004.tif]

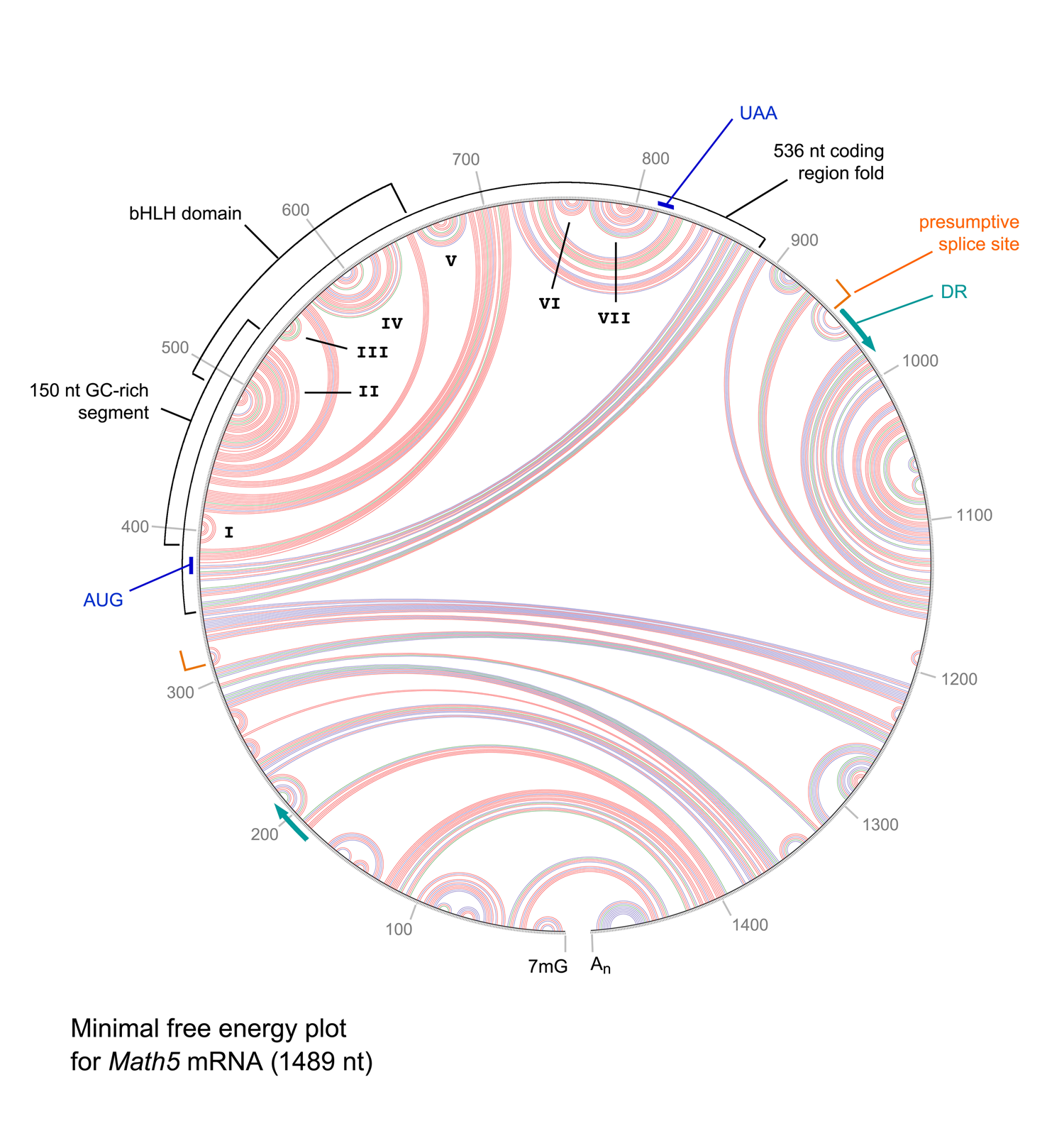

Supplement: Figure S5 — Secondary structure for Math5 mRNA. This circle plot was generated by free energy minimization of the 1489 nt mRNA, and is enlarged from Figure 7. Red, blue and green arc lines indicate G–C, A–U and A–G base pairs. The coding region, DRs and presumptive ECO splice sites are labeled. The 150 nt segment with >85% G+C, and 536 nt segment spanning the CDS are marked. The CDS contains a high density of G–C base pairs (red arcs), which are deleted in rare, mis-spliced RNAs. (1.33 MB TIF) [file pone.0012315.s005.tif]
