# Supplementary material for: A Critical Analysis of Atoh7 (Math5) mRNA Splicing in the Developing Mouse Retina
Source: PLoS One. 2010 Aug 24;5(8):e12315. doi: 10.1371/journal.pone.0012315 (PMC2927423; doi:10.1371/journal.pone.0012315)
Supplement: Table S1 — Oligonucleotide primers in this study. (0.03 MB PDF) [file pone.0012315.s006.pdf]

Table S1. Oligonucleotide primers in this study

Name	Ori	Sequence (5′→3′)	Expt	Alternate names‡	
				Sup. Fig. 2	Sup. Table 2
LP1	S	TCTACTGCAAGCTGTCCAAACGCTC	RT-PCR	1	
LP2	AS	AACATACAGGCTGTGTTGGTAGCTG	RT-PCR	2	
LP3	AS	GGTAGCTGCTCAGAACATAAACAAGTCACAT	RT-PCR	3	
LP4	AS	GTTTCTCCACCTCCTGAATGACGCT	RT-PCR, triplex PCR†	4	
LP5	S	GCCTCCCTATCTCCACTTCTCTTGTGT	RT-PCR, 3′RACE (pA1)	5	
LP6	S	GTGGATGAAGTCGGCCTGCAAA	RT-PCR	6	
LP7	AS	TTTCTCCCCTAAGACCCAAATGGC	RT-PCR	7	
LP8	S	TCTCAGGCTTTCCCAGAGAACTGGA	RT-PCR, triplex PCR		1
LP9	AS	TTTGCAGGCCGACTTCATCCAC	RT-PCR		2
AP	AS	GGCCACGCGTCGACTAGTACTTTTTTTTTTTTTTTTT	3′RACE		
UAP	AS	GGCCACGCGTCGACTAGTAC	3′RACE		
LP10	S	TCCCTATTGGGCGAAGTTGT	3′RACE (pA1)		
LP11	S	AGGGTGAAGTGCTTGCTGGT	3′RACE (pA6)		
LP12	S	GTTACAGGGCCTGCGAAATG	3′RACE (pA6)		
LP13	S	AAGCTGTCCAAGTACGAGACACTGC	RT-PCR, triplex PCR		
LP14	S	CCTTTTCTGCTTAATTTCCTTCCCG	triplex PCR		
LP15	S	GGGTGCTAGGCTCCAG | GTTTC	triplex PCR (Cb)*		

Notes:

Ori, orientation; S, sense; AS, antisense; AP, adapter primer; UAP, universal amplification primer

***primer sequence spans Cb intron junction**

†end-labeled with 6-carboxyfluorescein (6-FAM)

‡used by Kanadia and Cepko (2010)
