# Supplementary material for: A Critical Analysis of Atoh7 (Math5) mRNA Splicing in the Developing Mouse Retina
Source: PLoS One. 2010 Aug 24;5(8):e12315. doi: 10.1371/journal.pone.0012315 (PMC2927423; doi:10.1371/journal.pone.0012315)
Supplement: Table S2 — PCR conditions in this study. (0.03 MB PDF) [file pone.0012315.s007.pdf]

Table S2. PCR conditions in this study

Experiment	Primers FOR	Primers REV	# cycles†	Product	Size	Figure
RT-PCR (gPCR)	LP8	LP4	35§	Math5 full length<br>ECO	1087<br>448	3a, 3b, 4b
RT-PCR (gPCR)	LP6	LP7	35§		486	3c, 3d, 4c
RT-PCR	LP13	LP4	35	unspliced<br>Cb splice	567<br>368	Suppl 3d
RT-PCR	LP15	LP4*	33	Cb splice	228	Suppl 3e
3'RACE pA1	LP5<br>LP10	UAP<br>UAP	15<br>20	initial RACE<br>nested RACE	>591‡<br>>236‡	 2d
3'RACE pA6	LP11<br>LP12	UAP<br>UAP	15<br>20	initial RACE<br>nested RACE	>379‡<br>>342‡	 2d
Triplex RT-PCR¶	LP8<br>LP13	LP4*	33	unspliced<br>ECO	567<br>448	6b, 6c
Triplex RT-PCR¶	LP8<br>LP14	LP4*	33<br>33	unspliced<br>ECO	301<br>448	6b, 6c
Triplex RT-PCR¶	LP14<br>LP15	LP4*	33	unspliced<br>Cb splice	301<br>228	Suppl 3e

Notes:

* End-labeled with 6-FAM (carboxyfluorescein)

† All PCRs had an initial denaturation step (94°C x 3 min); followed by # cycles of 94°C x 30 sec denaturation, 57°C x 45 sec annealing, and 72°C x 60-70 sec extension; plus a final extension step (72°C x 7 min)

§ To generate deletion products, 40 cycles were used in these reactions, with basic *Taq* polymerase (Invitrogen cat. 10342)

‡ Products include a variable polyA tract

¶ Triplex PCRs utilized 3 primers, with 2 FOR primers at 0.1 µM each and 1 REV primer at 0.2 µM.
