# Supplementary material for: A Critical Analysis of Atoh7 (Math5) mRNA Splicing in the Developing Mouse Retina
Source: PLoS One. 2010 Aug 24;5(8):e12315. doi: 10.1371/journal.pone.0012315 (PMC2927423; doi:10.1371/journal.pone.0012315)
Supplement: Table S3 — DNA sequence flanking deletions in RT-PCR products. (0.04 MB PDF) [file pone.0012315.s008.pdf]

Table S3. DNA sequence flanking deletions in RT-PCR products

<table>
<tr><th>No.</th><th>5′ sequence</th><th>del</th><th>3′ sequence</th></tr>
<tr><td colspan="4">from Kanadia and Cepko (2010)</td></tr>
<tr><td>ECO</td><td>... ACTGACTGCAC|GTGAGTCGCTCCGTC</td><td>[639]</td><td>TGTTTTGTAG|GTTTCATGAGTGGACATC ...</td></tr>
<tr><td>InC</td><td>... CCCTCCGGCGGGAGCT|CGCGGCGCGC</td><td>[71]</td><td>|CGCGGCGCGCAGGCGTCTGGCGGCCAAC ...</td></tr>
<tr><td>E12P20</td><td>... TCGCGGCGCGCCCCCGT|GCGCGGCG</td><td>[85]</td><td>|GCGCGAGCGGCGCCGCATGCAGGGGCTG ...</td></tr>
<tr><td>32</td><td>... TCGCGGCGCGCCCCCG|TGCGCGGGCG</td><td>[134]</td><td>|TGCGCAGGGTGGTGCCGCAGTGGGGCCA ...</td></tr>
<tr><td>9</td><td>... GGCCCTCCGGCGGGAGCTCGC|GGCGC</td><td>[122]</td><td>GG|GGCTGAACACGGCGTTCGACCGGCTG ...</td></tr>
<tr><td colspan="4">from E14.5 retina RT-PCR (≤2X MasterAmp™, 40 cycles)</td></tr>
<tr><td>1</td><td>... AAGTCGGCCTGCAAACCCCAC|GGCCC</td><td>[70]</td><td>|GGCCCGGGCGGCTGGAGAGCGCGGCGCG ...</td></tr>
<tr><td>2*</td><td>... ATGAAGTCGGCCTGCAA|ACCCCACG</td><td>[235]</td><td>CTGCAG|ATGGCGCTCAGCTACATCATCG ...</td></tr>
<tr><td colspan="4">from RT-PCR of IVT RNA (≤2X MasterAmp™)</td></tr>
<tr><td>3</td><td>... CGTGGATGAAGTCGGCCTG|CAAACC</td><td>[746]</td><td>|TAATCCTAGCGTCATTCAGGAGGTGGA ...</td></tr>
<tr><td>4</td><td>... GAAAGGCTTTCTAT|CCCCGACCCCC</td><td>[605]</td><td>|CCCCTACCTCCCTTTCCCGGGTGCTAG ...</td></tr>
<tr><td>5</td><td>... CTCCGGCGGGAGCTC|GCGGCGCGCC</td><td>[106]</td><td>|GCGGCGCCGCATGCAGGGGCTGAACAC ...</td></tr>
<tr><td>6</td><td>... CTCCGGCGGGAGCTC|GCGGCGCGCC</td><td>[106]</td><td>|GCGGCGCCGCATGCAGGGGCTGAACAC ...</td></tr>
<tr><td>7</td><td>... CTCCGGCGGGAGCTC|GCGGCGCGCC</td><td>[70]</td><td>|CGCGGCGCGCAGGCGTCTGGCGGCCAA ...</td></tr>
<tr><td>8</td><td>... CGCGCCCCCGTGCGCGG|GCGCAGCC</td><td>[85]</td><td>GCGCGA|GCGGCGCCGCATGCAGGGGCT ...</td></tr>
<tr><td>9</td><td>... CTCGCGGCGCGCCCCCG|TGCGCGGGC</td><td>[103]</td><td>|TGCAGGGGCTGAACACGGCGTTCGACC ...</td></tr>
<tr><td>10</td><td>... CCCCACGGCCCTCCGGCGG|GAGCTCG</td><td>[110]</td><td>GCGC|GAGCGGCGCCGCATGCAGGGGCT ...</td></tr>
<tr><td>11</td><td>... CCCTCCGGCGGGAGCT|CGCGGCGCGC</td><td>[71]</td><td>|CGCGGCGCGCAGGCGTCTGGCGGCCAA ...</td></tr>
<tr><td>12</td><td>... TGCAAACCCCACGGCCCT|CCGGCGGG</td><td>[155]</td><td>|CCGGCTGCGCAGGGTGGTGCCGCAGTG ...</td></tr>
</table>

Spurious RT-PCR products are numbered to match Figure 5. The highlighted text shows areas of micro-homology, which can promote RT template switching. The size of the deletion [bp] and breakpoints (|) are indicated.

***Product is likely due to mis-priming of LP6.**
